# The therapeutic role of exercise training during menopause for reducing vascular disease

**DOI:** 10.1113/EP092191

**Published:** 2024-11-19

**Authors:** Conan L. H. Shing, Bert Bond, Kerrie L. Moreau, Jeff S. Coombes, Jenna L. Taylor

**Affiliations:** ^1^ Physiology and Ultrasound Laboratory in Science and Exercise (PULSE), Centre for Research on Exercise, Physical Activity and Health, School of Human Movement and Nutrition Sciences University of Queensland Brisbane Australia; ^2^ Public Health and Sport Sciences, Faculty of Health and Life Sciences University of Exeter Exeter UK; ^3^ Division of Geriatric Medicine, Department of Medicine University of Colorado Anschutz Medical Campus Aurora Colorado USA; ^4^ Eastern Colorado Health Care System Geriatric Research Education and Clinical Center Aurora Colorado USA

**Keywords:** arterial stiffness, cerebral blood flow, cerebrovascular function, hormone bioavailability, metabolic, oestrogen, vascular function

## Abstract

Menopause marks a major milestone in female reproductive ageing. It is characterized by the cessation of ovarian function and a concomitant decline in hormones such as oestradiol. Subsequently, females undergoing menopausal transition experience a progressive increase in cardiovascular and cerebrovascular disease risk. During menopause, reductions in nitric oxide (NO) bioavailability, endothelial dysfunction, increases in systemic inflammation, oxidative stress, and impaired vascular remodelling may contribute towards an accelerated decline in the function of cerebral and peripheral vascular systems. Historically, hormone therapy (HT) has been used as a means of managing vascular disease risk and reducing menopause‐associated vasomotor symptoms such as hot flushes, though some studies suggest regular exercise has the potential to be a promising alternative. Regular aerobic exercise during early postmenopause may slow vascular decline by improving NO and oestradiol bioavailability, promoting positive vascular remodelling and lowering systemic inflammation. However, exercise‐mediated improvements in markers of vascular function are not consistently observed in oestradiol‐deficient postmenopausal women. Emerging evidence suggests that due to the greater oestradiol bioavailability during early postmenopause, vascular adaptations to exercise may be enhanced during this stage, as opposed to late postmenopause. Subsequently it may be important to begin regular exercise in the years preceding and immediately following the final menstrual period to slow the progression of vascular disease risk during perimenopause and beyond. The present review will provide a summary of our current understanding of how vascular function is affected during menopause and the role of regular aerobic and resistance exercise training in managing vascular disease risk.

## Abstract (highlights)

- **What is the topic of this review?**
  This review focuses on the physiological changes that contribute to the accelerated risk of vascular disease throughout stages of menopause and the role that exercise may play in managing this risk.
- **What advances does it highlight?**
  This review acts as a summary of our current understanding of the therapeutic role of exercise throughout menopause and how it may contribute to both the management and the preservation of vascular health.

## 1 PREFACE

Although it is largely accepted that a 12‐month period of continuous amenorrhoea following the final menstrual period is defined as menopause, this might not be true for all women. This definition disregards external factors which may impact a woman's menstrual cycle and/or function. Secondary menopause may occur because of hysterectomy, endometrial ablation, or the use of intra‐uterine devices or oral contraceptives. However, for this review, menopause refers to the natural progression towards or the permanent and natural cessation of ovarian function in *cis*‐gendered women, and not menopause induced either pharmacologically or surgically. In addition, this review will not focus on women who go through menopause prematurely.

## 2 MENOPAUSE

**FIGURE 1:**
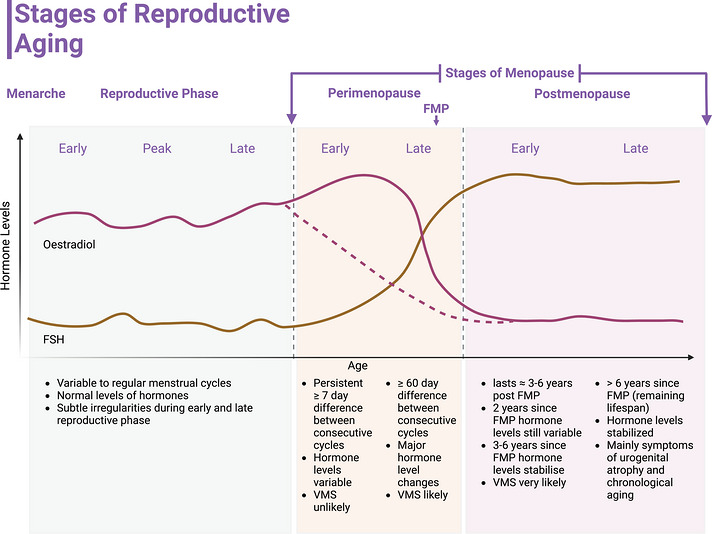
Stages of Reproductive Aging. Adapted from (Harlow et al., 2012) and (Tepper et al., 2012) with permissions from Oxford University Press on behalf of the Endocrine Society, Elsevier, Taylor & Francis, and Wolters Kluwer. Changes in oestradiol levels during perimenopause often present heterogeneously between women and although the transient increase during early perimenopause before a steep decline in late perimenopause is of one phenotype (depicted by a solid line), some women may experience a more progressive decline in oestradiol without the increase during early perimenopause (depicted by a dotted line). VMS: vasomotor symptoms, FMP: Final menstrual period, FSH: Follicle stimulating hormone. Created in BioRender. Shing, C. (2024) BioRender.com/u37t658.

Each year, approximately 25 million women worldwide enter menopause and experience its associated hormonal, physical, and psychological changes. Menopause is a significant milestone of female reproductive ageing, marking the cessation of regular menstrual cycles and a concomitant decline in ovarian function. The term ‘menopause’ is often used as an overarching term, encompassing both the several‐year‐long process before the final menstrual period and the years following it. Menopause consists of several stages, and the identification of each stage is dictated by the 2011 Stages of Reproductive Aging Workshop (STRAW+10) (Harlow et al., 2012). Figure 1 illustrates all the stages of reproductive ageing, including the reproductive phase (or premenopause), menopausal transition (otherwise called early/late perimenopause) and early/late postmenopause.

As the pool of ovarian follicles is depleted, the ability to produce and regulate essential hormones such as oestradiol, follicle‐stimulating hormone (FSH), and inhibin B becomes impaired (Burger et al., 1995). The three cardinal hormonal changes that characterize menopause are a gradual reduction in inhibin B levels, a progressive rise in FSH and a rapid decline in oestradiol 2 years before the final menstrual period (Figure 1) (Burger et al., 1995). However, the pattern of oestradiol depletion has been shown to be heterogeneous, with a slower, progressive decline throughout perimenopause seen in approximately 50% of women (Tepper et al., 2012). The transition to menopause can take approximately 3–9 years, with females often reporting a heterogeneous mix of both physical and psychological symptoms throughout the stages of menopause (Monteleone et al., 2018). Reported symptoms include, but are not limited to, hot flushes/night sweats (vasomotor symptoms), sleep disruption, depression, anxiety, vaginal dryness, decreased libido, increased urinary frequency, weight gain, increased abdominal adiposity, reduced muscle mass and hair loss (Monteleone et al., 2018). Notably, the severity and prevalence of these symptoms vary greatly, both between individuals and between stages of menopause (Figure 1). Therefore, while menopause is inevitable in women, the experience of menopause is individual.

## 3 CHANGES THAT OCCUR DURING MENOPAUSE AND THEIR ROLE IN VASCULAR DISEASE RISK

### 3.1 Endocrine changes

Each stage of menopause is associated with varying hormonal changes; however, the onset of menopause is often preceded by the progressive decline in two hormones, inhibin A and inhibin B (Øverlie et al., 2005). Both peptide hormones are part of a negative feedback loop that regulates the release of FSH and luteinizing hormone from the pituitary gland, making them inextricably linked and reliable indicators of ovarian ageing and menopausal status (Hall, 2015). As a woman enters perimenopause, previous levels of FSH are no longer able to readily stimulate the release of a follicle due to a reduction in the quality or number of eggs still available (otherwise known as diminished ovarian reserve). As a result, FSH levels continue to increase until a follicle is successfully released; at this point, inhibin B can once again regulate FSH levels (Hall, 2015). These feedforward mechanisms can be so powerful that during the menopausal transition, FSH levels can reach 10–15 times what is seen during the follicular phase in a woman's reproductive years (Hall, 2015).

The hormonal fluctuations and withdrawal that occur throughout the menopausal transition are thought to be the primary drivers behind why women experience menopausal symptoms and why women undergo a period of accelerated vascular decline during this phase of ageing (Monteleone et al., 2018; Moreau & Hildreth, 2014; Ryczkowska et al., 2023). However, whether other hormonal changes experienced during menopause are linked to vascular decline is not completely understood. In a cross‐sectional study of women across menopause stages, circulating FSH was more strongly correlated than oestradiol with the decline in brachial artery flow‐mediated dilatation (FMD) across menopause stages (Moreau et al. 2013), suggesting that an increase in FSH levels during the menopause transition may impact the vasculature more than fluctuations of oestradiol. In contrast, Aittokallio et al. did not find any association between FSH and inhibin B levels with impaired vascular function (Aittokallio et al., 2023). Whether changes in FSH, inhibin B, or other hormones contribute to the decline in vascular function warrants further investigation. In contrast, oestradiol levels are variable during perimenopause with a steady or rapid decline leading up to menopause, before plateauing and leaving postmenopausal women in a hypo‐oestrogenic state for their remaining lifespan (El Khoudary et al., 2020). Oestradiol is a powerful hormone that contributes to the control of many physiological processes beyond reproductive function, including cognition, bone health and cardiovascular health (El Khoudary et al., 2020; Genazzani et al., 2007; Khosla et al., 2012; Monteleone et al., 2018). The reduction in oestradiol levels during menopause may lead to changes in noradrenergic, serotonergic and dopaminergic signalling pathways within the central nervous system and are thought to contribute towards the increased prevalence of vasomotor symptoms, sleep disruptions, anxiety and depression (Bromberger et al., 2010; Monteleone et al., 2018; Woods & Mitchell, 2005).

### 3.2 Vascular changes

**FIGURE 2:**
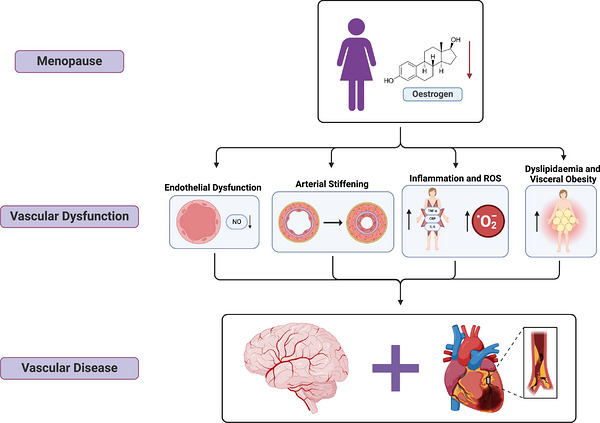
Mechanisms of vascular dysfunction during menopause: progressive reductions in NO bioavailability, large elastic arterial stiffening/thickening, systemic inflammation, increases in reactive oxygen species, dyslipidaemia and visceral obesity. The propagation of these mechanisms, in part, contributes to the progression and risk of systemic and cerebrovascular disease. NO, nitric oxide. Created in BioRender. Shing, C. (2024) BioRender.com/l12c784.

Vascular health is often delineated into two closely related factors: vascular function and vascular structure. Vascular function (also referred to as vascular reactivity) is the ability of the vessel to respond to changes in blood pressure, shear stress, or in response to physiological stressors such as exercise. Vascular structure refers to the physical characteristics and morphology of the vessel. Adverse changes in vascular structure and function include arterial stiffening and impaired endothelial function, which can often lead to the development of vascular diseases such as coronary artery disease, peripheral artery disease, stroke and vascular dementia (Figure 2).

A key driver behind the increased risk for vascular disease seen throughout menopause is the reduction in oestradiol levels. Emerging evidence supports that during the reproductive phase of a woman's life, oestradiol plays a cardioprotective role, maintaining vascular health, by regulating lipid metabolism, increasing angiogenesis, reducing fibrosis, promoting endothelial function, reducing oxidative stress, and regulating mitochondrial function (Ryczkowska et al., 2023). Hence, prior to menopause, women often have a lower risk of cardiovascular and cerebrovascular disease when compared to age‐matched men (Iorga et al., 2017). However, once a woman begins perimenopause and oestradiol levels begin to decline, the risk of vascular disease dramatically increases, often matching that of men by the time they reach postmenopause (El Khoudary et al., 2020).

#### 3.2.1 Endothelial dysfunction

The endothelium comprises primarily the nitric oxide (NO) and prostaglandin signalling pathways, which are responsible for endothelial‐mediated vasodilatation. Emerging research has shown oestrogens are important hormones that modulate vascular function. Within the endothelium, oestradiol, an isoform of oestrogen, has potent genomic and non‐genomic effects that influence several NO production pathways (Kypreos et al., 2014). The well‐known genomic pathway is regulated by oestradiol binding to α or β oestrogen receptors. Once bound, the new oestradiol‐bound oestrogen receptor complex (known as the oestrogen response element) now acts as a transcription factor responsible for upregulating endothelial nitric oxide synthase (eNOS) synthesis and subsequently NO production (Gliemann & Hellsten, 2019; Kypreos et al., 2014). A second independent non‐genomic pathway is also present and is known as the rapid signalling pathway. Similarly, this pathway is also mediated by the binding of oestradiol to either α or β oestrogen receptors. Once bound, the phosphoinositide 3‐kinase (PI3K)–protein kinase B (PKB or Akt) pathway activates eNOS to convert l‐arginine into NO via phosphorylation (Kypreos et al., 2014). From there, NO activates guanylate cyclase to begin producing cyclic‐guanosine monophosphate (GMP), triggering vascular smooth muscle‐mediated vasodilatation (Denninger & Marletta, 1999). In recent years, a third membrane‐bound receptor, G protein‐coupled receptor, has also been shown to interact with oestradiol to induce vasodilatation. Activation of this receptor triggers the same PI3K signalling cascade via a separate independent pathway mediated by the oestradiol‐induced activation of extracellular signal‐regulated kinase 1 and 2 (ERK1/2). However, this signalling pathway has been discovered relatively recently and further work is required to fully understand its role in regulating vascular function (Kypreos et al., 2014).

During menopause, reduced oestradiol levels may also adversely influence prostacyclin (PGI_2_), a potent prostaglandin responsible for regulating vascular function and vasodilatation alongside NO (Akarasereenont et al., 2000; Mendelsohn & Karas, 1994). Animal models suggest that following acute oestradiol withdrawal, the activity of vasoconstrictive prostaglandins (prostaglandin H synthase) is augmented, thereby increasing resting vascular tone. Furthermore, when postmenopausal women underwent acute oestradiol supplementation (2 mg of sublingual 17β‐oestradiol) PGI_2_‐mediated vasodilatation was enhanced, and synthesis of vasoconstrictive prostaglandins was reduced (Calkin et al., 2002).

During the transition to menopause, as oestradiol levels begin to decline, the production of both NO and PGI_2_ is reduced (Akarasereenont et al., 2000; Moreau & Hildreth, 2014). This subsequently leads to a reduction in vascular function reflected as a reduction, delay or uncontrolled vasodilatory response to changes in blood pressure or a physiological stressor. In postmenopausal women, this reduction in NO bioavailability is two‐fold; not only is there less oestradiol to activate eNOS (Moreau & Hildreth, 2014), but there are also fewer receptors for the remaining oestradiol to bind to (Gavin et al., 2009). Gavin and colleagues attributed the reduced oestrogen receptor expression to be in part due to the lower serum oestradiol concentrations during postmenopause. These findings were further supported when the same study compared oestrogen receptor expression in premenopausal women during the luteal (high hormone) and follicular (low hormone) phase of their menstrual cycle and found a similar correlation between oestrogen receptor expression and hormone concentration (Gavin et al., 2009).

A secondary mechanism that contributes to endothelial dysfunction during menopause is an increase in oxidative stress. Elevations in reactive oxygen species (ROS) can suppress eNOS activation, reducing NO availability and impairing endothelial function (Moreau & Hildreth, 2014). Interestingly, in oestradiol‐deficient environments, eNOS can become impaired and produce reactive oxygen and nitrogen species instead of NO; this process is referred to as eNOS uncoupling (Karbach et al., 2014; Moreau & Hildreth, 2014). Indeed the higher levels of oestradiol found in premenopausal women prevent this, acting as an effective antioxidant, inhibiting the production of ROS as well as actively scavenging ROS (Wagner et al., 2001). Moreover, postmenopausal women on long‐term hormone therapy (HT) (≥2 years of either oral or transdermal oestradiol, conjugated equine oestrogen, or combination progesterone/oestrogen) have presented with significantly greater endothelial function compared to those without HT (McCrohon et al., 1996). Taken together, these findings strongly support the beneficial role of oestradiol in regulating levels of ROS and endothelial function.

A third factor that contributes to endothelial dysfunction is vascular inflammation. Similar to oxidative stress, increased inflammation has been linked with impaired eNOS activation and elevated ROS production (Moreau & Hildreth, 2014). Furthermore, inflammation promotes leukocyte accumulation on the vascular wall, leading to the production of atherosclerotic plaques. In perimenopausal and early postmenopausal women (i.e., within 5 years of menopause), levels of inflammatory cytokines such as interleukin‐6 (IL‐6) were significantly elevated compared to premenopausal and late postmenopausal women (Bismar et al., 1995). Indeed women who chose to discontinue HT (ranging from 3 to 38 years of oral conjugated oestrogens or oestradiol) also presented with elevated levels of IL‐6 and tumour necrosis factor‐α (TNF‐α) when compared to both premenopausal and late postmenopausal women (Bismar et al., 1995). These findings suggest that not only is menopause associated with elevated inflammation, but oestradiol withdrawal appears to play a direct role in the concentration and production of pro‐inflammatory cytokines.

#### 3.2.2 Vascular structure

Adverse arterial stiffening is influenced by several structural components of the vessel, including the endothelium, smooth muscle, and the elastin and collagen found within the intima–media layer (Moreau & Hildreth, 2014). Although arterial stiffening increases relatively linearly with chronological ageing, during the 1 year before and after the final menstrual period, arterial stiffness steeply increases before plateauing again during the postmenopausal period (Samargandy et al., 2020). This time coincides with the greatest periods of oestradiol withdrawal and suggests that the declining oestradiol levels may augment age‐dependent arterial stiffening (Moreau & Hildreth, 2014; Samargandy et al., 2020). Indeed, Rajkumar et al. (1997) found that postmenopausal women who withdrew from HT (6.7 ± 1.0 years of oestrogen or combination estrogen‐progesterone therapy) for 4 weeks experienced a significant increase in arterial stiffness following 1 month of stopping HT. Furthermore, no differences in arterial stiffness were observed when comparing women using oestrogen‐only and combination progesterone–oestrogen therapy (Rajkumar et al., 1997). This study also demonstrated that oestradiol‐deficient postmenopausal women presented with greater arterial stiffness compared with premenopausal women (Rajkumar et al., 1997), although these changes may also be due to the significant age difference between the premenopausal (23 years old) and postmenopausal groups (58–59 years old) (Rajkumar et al., 1997). An observed increase in arterial stiffness suggests one or more of the mechanical components (elastin or collagen) responsible for the intrinsic elastic property of the vessels were impaired. However, the identification of changes in the microarchitecture of the central arteries requires highly invasive procedures (post‐mortem extraction of vessels), and therefore empirical measurements of vascular microarchitecture are largely limited to animal models (Chow et al., 2014; Latimer et al., 2014). As a result, it remains unknown how menopausal transition affects vascular microarchitecture in humans. Animal studies have demonstrated that oestradiol is able to promote elastin synthesis within the arterial wall (Matsuda et al., 1993), reduce collagen levels (Dubey, Jackson, et al., 2000) as well as limit smooth muscle proliferation (Dubey, Gillespie, et al., 2000; Yoon et al., 2001). Collectively, these findings suggest oestradiol deficiency experienced during the menopausal transition and beyond may significantly influence and alter vascular structure and microarchitecture.

In addition to changes in the micro‐architecture of the vascular wall, the proliferation of smooth muscle into the intimal space has also been shown to be a reliable marker of vascular disease progression. Specifically, increases in the thickness of the tunica media and tunica intima of the vessel wall, known as intima–media thickness, is indicative of subclinical atherosclerosis. Studies have demonstrated that women who reported long‐term HT use (>1 year of oestrogen‐only or combination oestrogen–progestin) presented with a lower intima–media thickness compared to women who had never used HT, suggesting oestradiol supplementation may also contribute towards the reduction of smooth muscle proliferation (McGrath et al., 1998; Moreau et al., 2002; Scuteri & Ferrucci, 2007; Tremollieres et al., 2000; Westendorp et al., 1999).

Collectively, these findings highlight the effects of oestradiol withdrawal during the menopausal transition and beyond in promoting adverse arterial stiffening and remodelling of vascular microarchitecture.

#### 3.2.3 Cerebrovascular dysfunction

Throughout menopause, dramatic alternations to the cerebral vasculature occur, affecting structure and function. Cerebrovascular function primarily refers to the ability of the cerebral vessels to regulate blood flow and ensure adequate perfusion to brain regions. In disease or ageing, poor cerebrovascular function is often reflected as either reduced cerebral blood flow at rest or impaired cerebrovascular reactivity (i.e., change in cerebral perfusion or cerebral artery velocity in response to vasoactive stimuli such as carbon dioxide or cognitive tasks).

Age‐related declines in cerebrovascular function have been linked to elevated central artery stiffness. Studies have reliably shown that with increases in aortic stiffness, excessive peripheral pulsatility results in damage to the delicate microvasculature of the brain. Indeed, several MRI studies have positively correlated increased peripheral pulsatility with microvascular brain lesions (Mitchell et al., 2011) and adverse changes to the microvascular structure of the brain (Iadecola & Davisson, 2008). Interestingly, studies have also demonstrated whole brain cerebral blood flow was greater in premenopausal women compared to age‐matched men (Guo et al., 2024). However, when comparing peri‐ and postmenopausal women to age matched men, this difference was abolished (Guo et al., 2024). This finding suggests that menopausal status may indeed contribute towards cerebrovascular dysfunction independent of ageing.

Investigations aimed at identifying the independent role of menopausal status in cerebrovascular decline have found that oestradiol may play a similar vasoprotective role in both the peripheral vasculature and the cerebral vasculature by promoting vasodilatation (Raz, 2014; Skinner et al., 2021). However, conflicting literature still exists surrounding the role of oestradiol and menopausal status on cerebrovascular function. One study demonstrated no difference in resting cerebral artery blood velocity and cerebrovascular reactivity between pre‐ and postmenopausal women (Ruediger et al., 2023). In contrast other studies have shown that oestradiol‐deficient postmenopausal women presented with reduced cerebral artery blood velocity and cerebrovascular conductance compared to premenopausal women (Brislane et al., 2020), and greater cerebral artery stiffness as measured by pulsatility index (Penotti et al., 1996). Notably, it is possible the observed reductions in cerebral artery blood velocity and cerebrovascular conductance are attributed to age and not oestradiol deficiency, with the average age of the postmenopausal women tested being 58.5 ± 5.5 years old (Penotti et al., 1996). Furthermore, conflicting literature also exists with respect to cerebrovascular reactivity to a carbon dioxide stimulus. One investigation found cerebrovascular reactivity was attenuated in both the intracranial (middle cerebral artery) and extracranial arteries (internal carotid artery) (Iwamoto et al., 2021) of postmenopausal women compared with premenopausal women, although another study reported reduced common carotid reactivity in postmenopausal women, but not intracranial cerebrovascular reactivity (Brislane et al., 2020). Currently, there is no clear consensus on precisely how menopausal status affects cerebrovascular function. Therefore, further longitudinal investigations are required to elucidate menopausal‐associated changes in cerebrovascular function independent of ageing.

In contrast, the role of HT in preserving cerebrovascular function appears to be clearer. Postmenopausal women who were taking HT (oestrogens) showed greater cerebrovascular reactivity compared to those who had not used HT (Kastrup et al., 1998). A meta‐analysis also found the pulsatility index improved with HT (Skinner et al., 2021). These findings support the hypothesis that oestradiol does indeed play a role in maintaining cerebrovascular function. Furthermore, as oestradiol levels decline during the menopausal transition, it is possible that the accelerated decline in cerebrovascular function is linked to declining oestradiol levels (Kastrup et al., 1998).

In recent years, a growing field of research has attempted to identify links between impaired cerebrovascular function and cognition. In non‐specific populations, it is believed that impaired cerebral blood flow may be associated with cognitive impairment (Fouda et al., 2019; Kim et al., 2021). However, studies acutely manipulating cerebral blood flow have yielded equivocal findings when investigating cognitive function (Ogoh, 2017). Thus, it remains unclear how cerebral blood flow affects cognition, and it remains unknown if menopausal status may contribute toward the development of cognitive impairment. A similar paucity of literature exists investigating the relationship between cerebral blood flow, reactivity and the psychological symptoms of menopause. Although some work has linked impaired regional blood flow to depression (Chithiramohan et al., 2022; Liao et al., 2017), these studies did not investigate menopausal status.

### 3.3 Metabolic changes

During the transition to menopause, studies show a transient increase in the prevalence of dyslipidaemia and visceral obesity (Figure 2) (Matthews et al., 2009). It is well known that dyslipidaemia and visceral adiposity are risk factors for the development of vascular diseases, such as atherosclerosis, coronary artery disease or stroke. Furthermore, both peri‐ and postmenopausal women often present with abnormal lipid profiles, such as elevated levels of total cholesterol, triglycerides and low‐density lipoproteins (LDL) (de Aloysio et al., 1999). Notably, studies have shown that although increases in blood pressure and body mass appear to reflect chronological ageing, changes in LDL and total cholesterol levels do not. Specifically, both LDL and total cholesterol increase steeply during the year preceding and following the final menstrual period before plateauing during postmenopause, coincident with the time of greatest oestradiol withdrawal (Matthews et al., 2009).

## 4 INFLUENCE OF EXERCISE ON VASCULAR HEALTH DURING MENOPAUSE TRANSITION

### 4.1 Effect of exercise on endothelial function

Acute exercise has been shown to manipulate endothelial function as measured by a change in brachial artery FMD in healthy young adults (Dawson et al., 2013). Studies investigating non‐specific populations show a biphasic FMD response. Immediately following aerobic exercise FMD is briefly attenuated followed by a normal or supranormal FMD in the following 24–48 h (Dawson et al., 2013). Similarly, studies that have investigated resistance training have demonstrated a decrease in FMD when tested within the first 30 min post‐exercise (Dawson et al., 2013). However, this reduction in FMD could be reflective of lasting increases in blood pressure and smooth muscle tone presenting as attenuated vascular function. Notably, both chronic aerobic and resistance training have reliably demonstrated improvements in the FMD response of healthy young adults (Green et al., 2004; Silva et al., 2021).

**FIGURE 3:**
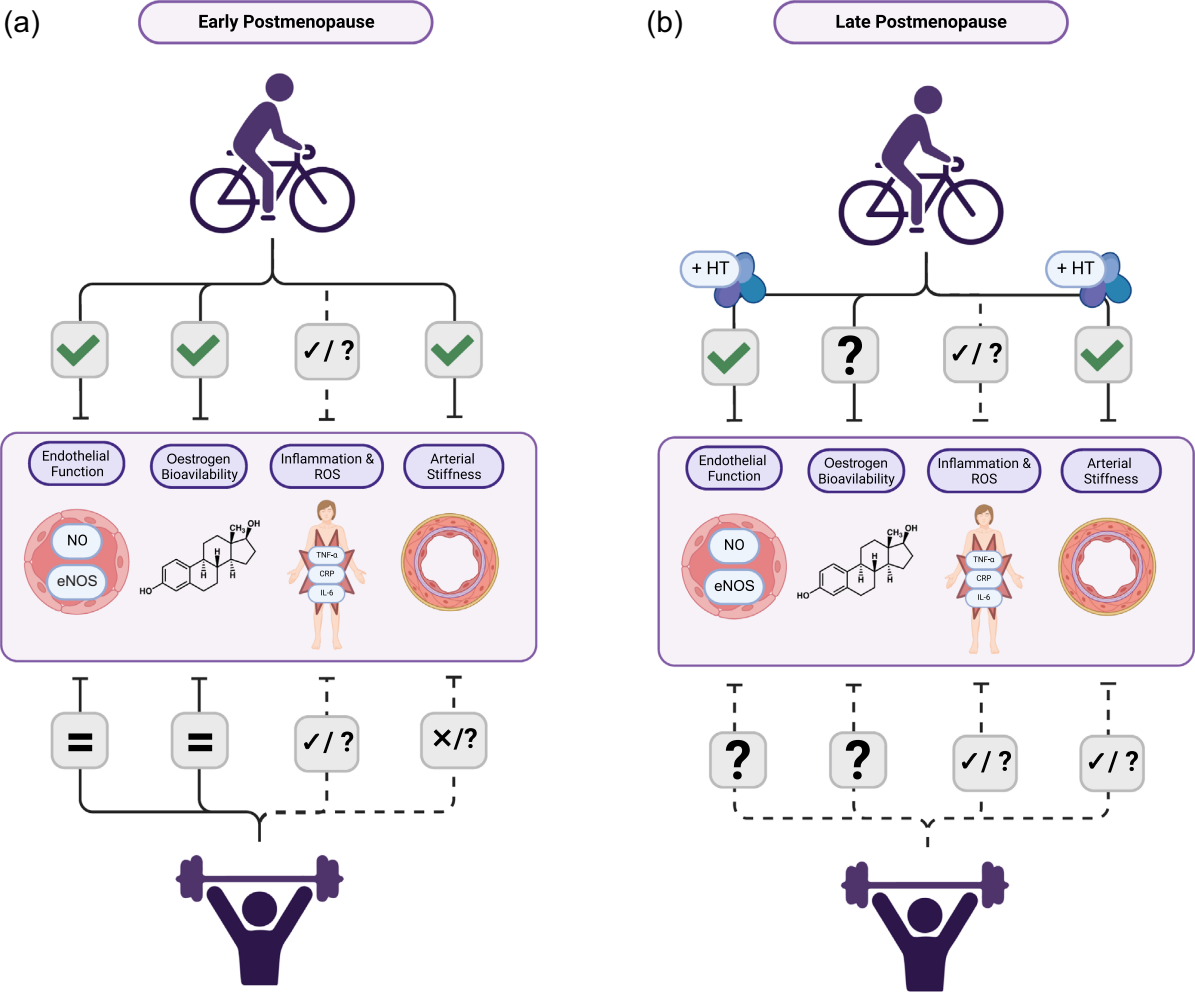
Effects of exercise training on indicators of vascular health during early postmenopause (a) and late postmenopause (b). Regular aerobic exercise during early postmenopause appears to improve all indicators of vascular health; however, during late postmenopause, some of these benefits require the use of HT to be revealed. In contrast, resistance exercise appears to potentially improve inflammation during both early and late postmenopause while potentially improving arterial stiffness only during late postmenopause. ‘✓’ indicates previous investigations support an improvement following exercise; ‘=’ indicates no change following exercise; ‘?’ indicates equivocal evidence for change following exercise ‘×/?’ indicates a potential adverse change following exercise, ‘✓/?’ indicates potential improvement following exercise; ‘HT’ indicates that hormone therapy use is required to elicit improvements following exercise or may augment exercise‐mediated effects. Created in BioRender. Shing, C. (2024) BioRender.com/b05c160.

In postmenopause, studies have demonstrated that a single bout of dynamic aerobic exercise produced conflicting responses in post‐exercise FMD. One study demonstrated that FMD improved to levels seen during premenopause following a single bout of aerobic exercise (45 min of treadmill exercise at 60% of maximal oxygen uptake (${\dot{V}}_{O_{2}\max}$)) (Harvey et al., 2005). Interestingly, this result contrasts with the biphasic response previously seen in healthy young adults when FMD is measured immediately following a single bout of aerobic exercise (Dawson et al., 2013). Additionally, the magnitude of post‐exercise FMD response was shown to be inversely related to baseline endothelial function in these postmenopausal women, whereby women who presented with greater initial impairment experienced a greater post‐exercise improvement in FMD (Harvey et al., 2005). When these same women underwent 4 weeks of 2 mg/day oral oestradiol supplementation, their FMD response was not further augmented following the same acute dynamic treadmill exercise. This suggests that oestradiol supplementation may not be additive to exercise‐mediated improvements in post‐exercise FMD (Harvey et al., 2005). In contrast, a separate investigation exposed postmenopausal women to a similar exercise bout (40 min of treadmill exercise at 60–75% of max heart rate) and demonstrated that FMD remained unchanged until participants were given a 0.05 mg/day transdermal oestradiol patch (Ozemek et al., 2020). Despite these conflicting results, the findings presented by Harvey et al. (2005) provided encouraging evidence behind the therapeutic potential of aerobic exercise in the absence of HT in oestradiol‐deficient postmenopausal women. Currently, it remains unknown how menopausal status may affect the acute FMD response to resistance exercise. Additionally, the effects of regular exercise training on endothelial function across the various stages of menopause remain inconclusive (Figure 3).

Currently, conflicting studies have demonstrated that chronic aerobic exercise can either improve endothelial function (He et al., 2022; Wenner et al., 2023) or has no effect on endothelial function in both early and late postmenopausal women (Moreau et al., 2013; Pierce et al., 2011). These discordant findings may be due to differences in methodology and vascular assessment techniques between investigations (Wenner et al. (2023) used strain gauge venous occlusion plethysmography, while the remaining studies utilized FMD). Studies that found no differences in endothelial function following endurance exercise training suggested that this was related, in part to low oestradiol bioavailability, possibly impairing exercise‐mediated vascular adaptations (Moreau et al., 2013; Pierce et al., 2011). In contrast, studies that demonstrated significant improvements in endothelial function following exercise training found these improvements were due to an increase in NO bioavailability (He et al., 2022) and a reduction in proteins responsible for vasoconstriction (endothelin 1) (Wenner et al., 2023). However, the degree of benefit experienced may also rely on both the type and the intensity of exercise performed. Despite regular resistance exercise training being an important component of weekly physical activity guidelines, its effect on endothelial function in postmenopausal women remains understudied (Moreau et al., 2024). In summary, the effects of resistance exercise training on endothelial function in postmenopausal women are unclear and regular endurance exercise does not appear to consistently improve endothelial function in postmenopausal women without HT, as summarised in Figure 3. Furthermore, to our knowledge, no study has directly investigated the effects of exercise training during perimenopause, and it is unclear whether vascular adaptations to exercise differ during this phase of menopause.

When comparing the mechanisms behind exercise‐mediated vascular adaptations across stages of menopause, improvements observed in healthy early postmenopausal women (Nyberg et al., 2016) or age‐matched men are not consistently observed in late postmenopausal women (Gliemann & Hellsten, 2019). The upregulation of NO production pathways observed following endurance exercise in early postmenopausal women is thought to be maintained through a combination of oestradiol, muscle contraction and shear stress‐mediated pathways (Gliemann & Hellsten, 2019). Interestingly, some studies have also demonstrated that following acute bouts of exercise, oestradiol production is transiently augmented in postmenopausal women. These increases in oestradiol may potentially activate the genomic and non‐genomic NO production pathways (Gliemann & Hellsten, 2019) (discussed in Section 3.2.1). However, previous studies have demonstrated that irrespective of time since menopause, both early and late postmenopausal groups were unable to improve their FMD response (Moreau et al., 2013; Pierce et al., 2011). The reasons behind these discrepant findings are not completely understood. However, it has been speculated that changes in oestradiol levels and oestradiol receptor expression may be related to time after menopause and could influence the effects of exercise training in postmenopausal women (Gliemann & Hellsten, 2019; Nyberg et al., 2016). Consistent with this, endothelial function, as measured by FMD, was enhanced in healthy early and late postmenopausal women treated with oestradiol therapy but not in women who were given a placebo treatment and who remained oestradiol‐deficient (Moreau et al., 2013). It is also possible that differences in the prescribed exercise intensity may also play a role independent of oestradiol bioavailability and time since menopause. Nyberg et al. (2016) saw improvement with a 12‐week 3 days/week 1‐h cycle at 70%–95% max heart rate whereas Moreau et al. (2013) found no improvement with a 12‐week 6–7 days/week 40–50 min run at 70–75% max heart rate.

Evidence that oestradiol bioavailability might influence exercise adaptations gave rise to the potential of a timing effect for exercise training in women undergoing menopausal transition (Gliemann & Hellsten, 2019). Gliemann and Hellsten suggested that if exercise training occurs during early postmenopause, oestradiol and oestradiol receptor expression levels may still be sufficient to elicit exercise‐mediated adaptations of vascular function. Furthermore, evidence suggests that progressive age and oestradiol withdrawal‐associated increases in low‐grade inflammation and elevated ROS may counteract any potential exercise‐mediated improvements in NO production/function. This highlights the importance of starting exercise training early during menopause. Additionally, this may be a reason that oestradiol‐deficient late postmenopausal women experience equivocal improvement in endothelial function following exercise training (Gliemann & Hellsten, 2019; Moreau et al., 2013). However, existing literature investigating this hypothesis has yielded equivocal findings, so although time since menopause appears to influence exercise‐mediated vascular adaptations, further studies are warranted to identify its role.

### 4.2 Arterial stiffness

Age‐related arterial stiffening or reduction in arterial compliance is an inevitable physiological process, with menopause marking a period of accelerated vascular decline (Samargandy et al., 2020; Tanaka et al., 1998). Studies have suggested regular aerobic exercise may be a reliable strategy for preserving arterial compliance in mid‐life and older women (Tanaka et al., 1998). Previous investigations have consistently demonstrated that without HT, endurance‐trained postmenopausal women present with greater carotid arterial compliance compared to their sedentary late postmenopausal counterparts (Figure 3b) (Moreau et al., 2003). Notably, these exercise‐trained women in the study had engaged in running exercise for an average of 5 days a week for the past 5 years. As a result, it is possible that the reason arterial compliance was maintained is due to habitual exercise performed before, during and after the menopausal transition. This study further demonstrated that aerobic exercise and chronic HT use appear to also have an additive effect on improving carotid arterial compliance in previously sedentary women (Moreau et al., 2003). Chronic HT use was defined as women who have undergone 10+ years of oral or transdermal conjugated oestrogens, oestradiol, or a combination of oestrogen and progestin forms of HT. When these participants were exposed to a 3‐month brisk walking exercise intervention, measures of arterial stiffness improved to levels of premenopausal women (Moreau et al., 2003). These findings suggest that HT use (in addition to exercise) may be necessary to fully recover measures of arterial stiffness to levels seen in young, healthy individuals (Moreau et al., 2003). However, to our knowledge, no study has directly compared the effect of exercise on arterial stiffness in current HT users and non‐HT users, and further investigations are needed to understand the role of HT further. In addition, it remains unknown how acute aerobic exercise may affect measures of arterial stiffness in menopausal populations.

Several mechanisms may be responsible for the observed improvements in arterial compliance following acute aerobic exercise and short‐term aerobic exercise training: (1) short‐term NO‐mediated relaxation of vascular smooth muscle tone improving arterial compliance (Moreau et al., 2003), (2) augmented endothelial function (discussed above), or (3) reduced oxidative stress (Moreau et al., 2006). Additionally, exercise and oestradiol‐mediated structural changes may also contribute to improvements in arterial compliance. Following chronic exercise or oestradiol supplementation, a reduction in collagen synthesis and an increase in elastin content within the arterial walls have been observed in rat models (Matsuda et al., 1993). However, in humans, it is likely these structural changes would take several years and it is unlikely the short‐term exercise studies discussed would produce changes in vascular microarchitecture (Moreau et al., 2003).

A dearth of knowledge exists regarding the effects of resistance exercise, independent of aerobic exercise, on arterial stiffness, particularly in perimenopausal populations. A meta‐analysis and several more recent studies have demonstrated that at least using combined resistance and aerobic training, postmenopausal females were able to improve their brachial‐ankle pulse wave velocity (Liu et al., 2023; Manojlović et al., 2021; Xi et al., 2021). One study showed that when sedentary postmenopausal women underwent 18 weeks of high intensity full body resistance exercise, no improvement in central aortic pressure wave reflection was observed (Casey et al., 2007). Interestingly, some studies have demonstrated regular resistance exercise may instead lead to increases in arterial stiffness in young healthy women (Cortez‐Cooper et al., 2005). However, these findings have not been replicated in postmenopausal women, and currently it appears resistance exercise does not consistently improve arterial stiffness in postmenopausal women (Figure 3). Furthermore, it remains unknown whether the acute responses to resistance exercise are different in peri‐ or postmenopausal women, when compared to premenopausal females or healthy young males.

### 4.3 Cerebrovascular function

Similar to the peripheral vasculature, regular aerobic exercise is beneficial for preserving and maintaining cerebrovascular function. Indeed, higher aerobic fitness has been positively correlated to improved cerebrovascular conductance, particularly in older adults (Brown et al., 2010). A previous investigation demonstrated that exercise‐trained postmenopausal women had preserved cerebrovascular function compared to age‐matched sedentary counterparts. These improvements were reflected as an increase in cerebrovascular conductance at rest, during/following exercise, and in response to carbon dioxide (Brown et al., 2010). Furthermore, a pilot study of inactive late postmenopausal women showed improvements in intracranial artery (middle cerebral artery) velocity and reactivity to carbon dioxide following 12 weeks of supervised high‐intensity interval training (Northey et al., 2019). Notably, this involved small sample sizes and the inclusion of women previously diagnosed with breast cancer. It is unknown how the previous diagnosis of breast cancer may have affected these findings and if larger‐scale trials would be able to replicate these findings.

There have been a few attempts at identifying the mechanisms behind the effects of exercise on cerebrovascular function. However, none directly investigated its role in peri‐ or postmenopausal populations. A recent study showed that with acute cycling exercise, exercise‐induced increases in internal carotid artery shear improved internal carotid artery reactivity and dilatation responses (Sakamoto et al., 2024). This finding is encouraging and suggests that, at least acutely, exercise can augment cerebrovascular function (Sakamoto et al., 2024).

The effect of resistance exercise on cerebrovascular function in middle‐aged and older women remains unclear. One study demonstrated that, compared to men, women exhibited greater increases in resting middle cerebral artery velocity immediately following acute high‐intensity resistance exercise (Marôco et al., 2023), although women had lower increases in carotid pulsatility and similar increases in MCAv pulsatility to men. However, this study did not compare cerebrovascular reactivity following exercise, and it remains unknown if any changes in cerebrovascular function were present immediately following resistance exercise. In another investigation, when healthy young adults were exposed to 3 months of whole‐body resistance training no improvements in any domains of cerebrovascular reactivity were observed, though measures of cerebrovascular resistance of the intracranial (middle, posterior) and extracranial (internal carotid) arteries were elevated (Thomas et al., 2021). As the latter investigation was conducted in healthy young adults, it remains unknown how resistance exercise affects cerebrovascular function across stages of menopause.

Despite several studies showing the beneficial effects of exercise on peripheral vascular function, there remains a paucity of literature investigating its role within the cerebrovasculature. As such, future longitudinal studies should be done to identify whether similar benefits are observed and if the mechanisms responsible for peripheral vascular remodelling and maintenance of endothelial function also exist within the cerebrovasculature during the menopausal transition and beyond.

### 4.4 Hormonal bioavailability

HT has traditionally been used as a means of managing both cardiovascular disease risk and menopause‐associated vasomotor symptoms, such as hot flushes and night sweats. Although HT is a reliable treatment for managing menopausal symptoms, it may also expose users to unwanted side effects. As a result, the use of exercise and physical activity as an alternative to HT has been gaining popularity in recent years. Indeed, previous work has shown that when women partake in regular exercise, vasomotor symptoms appear to improve throughout the menopausal transition and beyond (Daley et al., 2007), although studies have also shown that exercise may potentiate the severity of vasomotor symptoms (Romani et al., 2009).

Though the mechanisms behind the health benefits of exercise are multifaced, the transient increase of oestradiol levels both during and immediately following exercise has been posited to be a key driver behind these improvements. A previous investigation had demonstrated that irrespective of age (19–69) and exercise modality (endurance *vs*. resistance), oestrogen levels are significantly augmented immediately following exercise before returning to baseline levels after 30 min (Copeland et al., 2002). Similarly, a second investigation demonstrated that serum oestradiol levels were 20% higher than baseline for upwards of 120 min following 65 min of combined endurance, strength and jumping exercises in early postmenopausal women (Figure 3a) (Kemmler et al., 2003).

In contrast, chronic strenuous endurance training in young women has been well studied, and it appears to have detrimental effects on reproductive health (Cho et al., 2017; De Souza et al., 2010). A previous investigation demonstrated that in young untrained women, 8 weeks of strenuous endurance training significantly reduced serum oestradiol (Bullen et al., 1985). Although it is unlikely that recreationally active women will experience bouts of training strenuous enough to dramatically affect oestradiol levels, it suggests there may be an optimal dose of exercise for maximizing increases in oestradiol bioavailability. However, it remains unknown if chronic aerobic or resistance exercise may elicit long‐lasting changes in oestradiol bioavailability across menopausal populations.

As discussed above, previous work has shown that HT use may be necessary in postmenopausal women to elicit the greatest vascular improvements in previously sedentary women (Moreau et al., 2013). However, it remains unclear if the transient increases in oestradiol following exercise are potent enough to elicit improvements in vascular function during the earlier stages of the menopausal transition and beyond. It also is unclear whether a dose–response relationship exists whereby a longer or more intense exercise bout may trigger greater and longer increases in oestradiol. As a result, future studies should investigate this potential dose–response relationship, incorporating longer sampling periods (i.e., 4 h post‐exercise) to identify the true time course of augmented oestradiol production following exercise.

### 4.5 Anti‐inflammatory and metabolic contributions

During the transition to menopause, the withdrawal of oestradiol has been associated with increased systemic inflammation (Bismar et al., 1995). The accelerated increase in inflammatory cytokines during menopause such as TNF‐α, C‐reactive protein and IL‐6 may also predispose women to chronic diseases such as cardiovascular disease, metabolic syndrome or type 2 diabetes (Khalafi et al., 2021). Interestingly acute vigorous or high‐intensity exercise often results in a transient increase in inflammatory markers (Cerqueira et al., 2020), though over time and with adequate recovery, regular exercise training has been shown to reliably reduce chronic inflammation, improve body composition and augment metabolic function in postmenopausal women (Bueno‐Notivol et al., 2017; Khalafi et al., 2021; Nicklas & Brinkley, 2009; Tan et al., 2023; Yeh et al., 2018).

A key mechanism behind the anti‐inflammatory and metabolic benefits of exercise may be driven by reductions in both fat mass and visceral adipose tissue following regular aerobic training (Woods et al., 2011; Yeh et al., 2018). Adipose tissue inflammation is caused by dysfunctional adipocytes, which secrete inflammatory adipokines, signalling the production of pro‐inflammatory cytokines (Kawai et al., 2021). As a result, reductions in fat mass and adipose tissue, particularly visceral adipose tissue, have been shown to reduce both levels of inflammatory markers and the risk of cardiometabolic disease in postmenopausal women (Woods et al., 2011). A meta‐analysis in postmenopausal women showed that 3–4 months of regular aerobic or resistance exercise lowered insulin levels, body mass index, waist circumference, and percentage body fat during postmenopause (Bueno‐Notivol et al., 2017). Though this meta‐analysis did not measure inflammatory markers, it is reasonable to assume, that as body fat and body composition significantly improved, so would levels of inflammatory markers. Resistance training has been shown to reduce inflammation via a separate independent pathway by reducing skeletal muscle expression of TNF‐α (Greiwe et al., 2001; Sardeli et al., 2018). Thus, two distinct pathways may work additively to reduce systemic inflammation. Additionally, a meta‐analysis in women during post‐menopause showed that either aerobic, or resistance or combined training is effective in reducing pro‐inflammatory cytokines, although combined exercise showed the greatest reduction (Figure 3) (Khalafi et al., 2021). In addition, it showed that improvements in pro‐inflammatory cytokines did not differ between younger (age <64 years) and older postmenopausal women (age ≥64 years) (Khalafi et al., 2021). However, this investigation did not clearly separate early and late postmenopausal women, and it remains unclear how menopausal status may affect the above‐mentioned exercise‐mediated pathways. However, as emerging evidence continues to show the role of oestradiol deficiency in promoting inflammation, it may be important to emphasize incorporating regular exercise throughout menopause and beyond (Au et al., 2016).

## 5 CURRENT EXERCISE GUIDELINES AND RECOMMENDATIONS

### 5.1 Exercise recommendations during menopause

Exercise has long been the cornerstone of lifestyle modifications for managing chronic disease risk and progression. As discussed above, the menopausal transition can accelerate cardiovascular disease risk (El Khoudary et al., 2020) and age‐related vascular decline, and exercise may be a reliable treatment to slow the progression of vascular dysfunction (Moreau et al., 2024). As a result, it is important to incorporate regular exercise training early in the menopausal transition to help manage risks associated with ageing and, more importantly, the accelerated vascular disease risk experienced during the menopausal transition (El Khoudary et al., 2020).

Current adult global physical activity guidelines (Bull et al., 2020) from The World Health Organisation recommend at least 150–300 min of moderate‐intensity aerobic activity or 75–150 min of vigorous aerobic activity weekly. In addition, it is recommended to partake in at least 2 or more days of strength/resistance training every week. The American College of Sports Medicine also recommends incorporating a variety of exercises such as aerobic, resistance, neuromotor or flexibility exercises during menopause (Perez & Garber, 2011). Statements from the International Menopause Association recommend at least 150 min of moderate‐intensity aerobic activity and incorporating at least 2 or more days of strength/resistance training every week. These recommendations are in line with general adult physical activity guidelines, and it does not appear that menopause status affects these global exercise recommendations. As such, women during menopause can use these guidelines to set their weekly activity targets. Despite this, practitioners should ensure they are using a stepwise approach. Slowly increasing the workload and intensity to reduce exercise‐related injuries may be particularly important in previously sedentary women (Mishra et al., 2011).

Notably, HT may not be necessary to elucidate certain exercise‐induced improvements in vascular function and fitness (Figueroa et al., 2003). Although some studies have found that HT when used in conjunction with exercise training can augment muscle performance and composition (Sipilä et al., 2001), as well as exercise capacity (Mercuro et al., 2007), several other studies have shown the opposite in postmenopausal women. These studies demonstrated that although HT can improve measures of vascular function (Moreau et al., 2013), measures of cardiorespiratory fitness (Stathokostas et al., 2008) and strength (Figueroa et al., 2003) were not affected by HT use. Although exercise used in conjunction with HT may elicit the greatest improvements in vascular function, no studies have demonstrated that HT alone is superior to exercise.

## 6 CURRENT GAPS IN RESEARCH

Currently, exercise recommendations for postmenopausal women or women undergoing perimenopause align well with general global physical activity guidelines. However, no specific guidelines exist for menopausal populations, and it remains unknown if certain exercise intensities may elucidate greater and more time‐efficient improvements in vascular health in these groups. Furthermore, the role of resistance training in augmenting vascular function remains understudied, particularly in women undergoing menopausal transition and during postmenopause (Moreau et al., 2024).

Many of the existing studies investigating the effect of exercise on vascular function during menopause present contradictory findings and leave our current understanding of how exercise affects vascular function during menopause unclear. Future trials should be conducted to identify how varying exercise modalities and exercise intensities can independently affect vascular adaptations and whether the mechanisms behind these adaptations are parallel between the peripheral and cerebral vasculature throughout stages of menopause. Moreover, the effect of exercise on vascular health in perimenopausal women remains unknown. To our knowledge, no studies have directly investigated the effects of exercise training in perimenopausal women. As a result, it is unclear whether the mechanisms and pathways responsible for exercise‐mediated vascular adaptations are similar during perimenopause.

## 7 CONCLUSION

Menopause marks a period of significant and accelerated cardiovascular disease risk in women. The increased risk of vascular disease may be attributed to declining levels of oestradiol as women progress through the menopausal transition and beyond. Oestradiol deficiency may affect the vasculature directly, though its effect is global, impacting systemic inflammation and metabolic function. Some evidence suggests that exercise may be a reliable and potent intervention, whereby improving endothelial function, hormonal bioavailability and systemic inflammation slows down the progression of vascular disease risk during menopause. Subsequently, it is important to exercise early and often during the years preceding and during the menopausal transition, where oestradiol levels may still be high enough to elucidate vascular adaptations to exercise.

## AUTHOR CONTRIBUTIONS

Conan L. H. Shing was responsible for the drafting of the initial manuscript. Critical revision of important intellectual content was done by all authors. All authors have read and approved the final version of this manuscript and agree to be accountable for all aspects of the work in ensuring that questions related to the accuracy or integrity of any part of the work are appropriately investigated and resolved. All persons designated as authors qualify for authorship, and all those who qualify for authorship are listed.

## CONFLICT OF INTEREST

None declared.
